# Succinate-Directed Approaches for Warburg Effect-Targeted Cancer Management, an Alternative to Current Treatments?

**DOI:** 10.3390/cancers15102862

**Published:** 2023-05-22

**Authors:** Adrian Casas-Benito, Sonia Martínez-Herrero, Alfredo Martínez

**Affiliations:** Angiogenesis Group, Oncology Area, Center for Biomedical Research of La Rioja (CIBIR), 26006 Logroño, Spain

**Keywords:** cancer, metabolism, Warburg effect, glucose, glycolysis, lactate, succinate

## Abstract

**Simple Summary:**

One hundred years ago, it was discovered that some cancer cells have a different metabolism from normal cells. This alternative metabolism is called the Warburg effect, and instead of using glucose to produce energy through the oxidative pathway, the cancer cells undergoing this effect use fermentation, meaning that they behave just as cells exposed to hypoxia do. Despite the fact that it provides less energy, the advantage of using the Warburg metabolism is that cells produce and/or accumulate large quantities of intermediates that favor cell division and tumor progression. Succinate is one of these intermediates, which is accumulated in larger amounts in the Warburg context and contributes to cancer growth. In this review, succinate’s protumoral characteristics are described, and its target value to develop alternative treatment for cancer is discussed.

**Abstract:**

Approximately a century ago, Otto Warburg discovered that cancer cells use a fermentative rather than oxidative metabolism even though the former is more inefficient in terms of energy production per molecule of glucose. Cancer cells increase the use of this fermentative metabolism even in the presence of oxygen, and this process is called aerobic glycolysis or the Warburg effect. This alternative metabolism is mainly characterized by higher glycolytic rates, which allow cancer cells to obtain higher amounts of total ATP, and the production of lactate, but there are also an activation of protumoral signaling pathways and the generation of molecules that favor cancer progression. One of these molecules is succinate, a Krebs cycle intermediate whose concentration is increased in cancer and which is considered an oncometabolite. Several protumoral actions have been associated to succinate and its role in several cancer types has been already described. Despite playing a major role in metabolism and cancer, so far, the potential of succinate as a target in cancer prevention and treatment has remained mostly unexplored, as most previous Warburg-directed anticancer strategies have focused on other intermediates. In this review, we aim to summarize succinate’s protumoral functions and discuss the use of succinate expression regulators as a potential cancer therapy strategy.

## 1. Introduction

Cancer is a generic term for a large group of diseases characterized by the uncontrolled proliferation of cells that can affect any part of the body. According to Globocan data, in 2020, there were more than 19 million new cancer cases diagnosed worldwide, and the 5-year prevalence for this disease was accounted in 50.5 million cases [1]. The rapid creation of abnormal cells that grow and multiply beyond their normal boundaries not only affects the main organ where the tumor is originated, but also can invade adjoining organs or spread to other parts of the body. In fact, widespread metastases are the primary cause of death from cancer [1].

The World Health Organization has established that cancer is now the leading cause of death worldwide, accounting for nearly 10 million deaths in 2020 [2]. Cancer treatment usually includes surgery, radiotherapy, and/or systemic therapy (chemotherapy, hormonal treatments, or biological therapies) [3,4,5,6]. Proper selection of a treatment regimen takes into consideration both the cancer and the patient being treated. The primary goal of anti-cancer therapies is generally to cure cancer and/or to prolong life. However, we cannot forget that improving the patient’s quality of life is also an important aim. Although many efforts have been made to achieve improvements to reduce the occurrence of anti-cancer therapy side effects, most patients still experience burdensome effects after receiving any of the current treatments available [4,7,8,9]. The patients and their families are deeply impacted by side effects in their everyday lives, and in many cases, these effects can be long-lasting [10].

Given this background, it is clear there is an urgent need for new tools in the fight against cancer not only to improve patients’ survival, but also to limit side effects and improve the quality of life, wellbeing, and mental health state of the patients and their families. Most of the times, side effects are caused because the treatment options cause the death not only of tumor cells, but also of normal cells of the body [4,7,8,9]. By targeting more specific features of cancer cells that are not shared with non-tumorigenic cells, we could improve treatment efficacy by reducing side effects at the same time.

That is why a better understanding of unique cancer hallmarks through a deep cancer biology characterization is needed to find new therapeutic approaches that help to improve survival and patients’ wellbeing. During the last decades, one of those well-stablished cancer hallmarks has started to gain more attention in the cancer research field, namely the metabolic reprogramming that occurs on cancer cells, also known as the Warburg effect. In fact, the number of publications about the Warburg effect on Pubmed is around 50 times higher than it was twenty years ago.

In this review, we summarize the main features that occur in the cell metabolism of most cancer cells, which are also essential for cancer progression. We have analyzed the metabolic reprogramming of cancer cells with an especial focus on the Warburg effect and its related oncometabolites, such as succinate. Although the Warburg effect research has exponentially increased in the past years, its potential as a new target for anti-cancer therapies development has been scarcely explored. The same is happening with succinate, a metabolite which is known to be accumulated on cancer metabolism and has protumoral functions. Here, we intend to summarize the current knowledge and gaps on the role of the Warburg effect and discuss possible alternatives and new research avenues based on succinate or other dysregulated parts of the Warburg metabolism and how they can be used in anti-cancer research.

## 2. Warburg Effect

### 2.1. Cancer Cells Have a Different Metabolism

The main metabolic pathway used by most cells to obtain energy is oxidative phosphorylation, which includes the Krebs cycle, as it is the most efficient way to produce energy in the form of adenosine triphosphate (ATP). Glucose is the main biochemical fuel, which enters the cells through glucose transporters (GLUT) and undergoes glycolysis, providing pyruvate as a final product. Under normoxic conditions, although a small amount of pyruvate is transformed into lactate, most of it enters the mitochondrion where it is converted into acetyl-CoA, which is the main substrate for the citric acid cycle (CAC), also known as tricarboxylic acid cycle (TCA) or Krebs cycle. The main function of the TCA is the production of reducing power in the form of nicotinamide adenine dinucleotide (NADH) or flavin adenine dinucleotide (FADH2). These reduced molecules are the main electron donors for the mitochondrial electron transport chain (ETC) which generates more than 30 ATP molecules per molecule of glucose, making it the most important and efficient source of energy in the cell [11].

**Figure 1:**
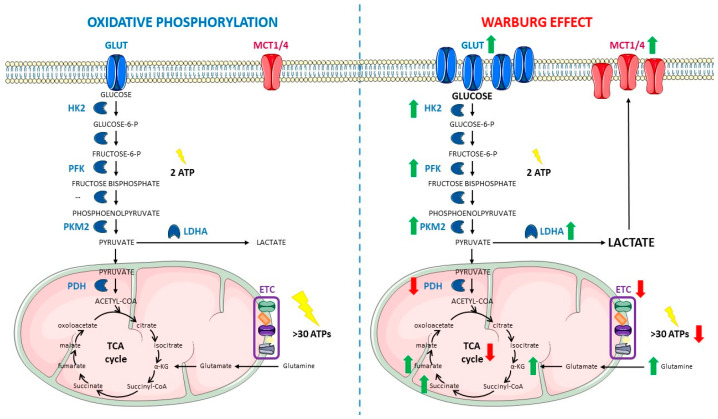
Comparison between oxidative phosphorylation (**left**) and the Warburg effect (**right**). Within oxidative metabolism, glucose is introduced inside the cell by Glucose Transporters (GLUTs) and degraded through glycolysis to provide pyruvate, which is mainly introduced inside the mitochondrion, with only a small amount being converted into lactate in the cytoplasm. Inside the mitochondrion, acetyl-CoA is obtained from pyruvate and enters the Tricarboxylic Acid (TCA) cycle to generate intermediates for the Electron transport Chain (ETC). Glutamine can be an important source of TCA α-ketoglutarate (α-KG) by entering inside the mitochondrion, being converted into glutamate and finally into the TCA intermediate. The whole process allows the cell to obtain more than 30 Adenosine Triphosphate (ATP) molecules per each glucose molecule catabolized. Under Warburg metabolism, GLUTs are overexpressed, which results in an increased glucose uptake. At the same time, there is an upregulation in the glycolytic enzymes Hexokinase 2 (HK2), Phosphofructokinase (PFK), and Pyruvate Kinase M2 isoform (PKM2), which leads to an increased glycolytic rate. The glycolytic rate may exceed the mitochondrial rate of pyruvate oxidation, thus making lactate accumulation unavoidable when glucose is so abundant inside the cells. Furthermore, Lactate Dehydrogenase A (LDHA) is also overexpressed in cancer metabolism, contributing to the generation of high levels of lactate. This accumulated lactate is pumped out of the cells through Monocarboxylate Transporters (MCT1/4) causing microenvironment acidification, which in turn triggers protumoral signaling pathways. In these conditions, Pyruvate Dehydrogenase (PDH) and the TCA cycle are also downregulated. At the same time, in several types of cancer, the coincidence of several mutations in the TCA cycle enzymes and the decreased entry of pyruvate into the TCA cycle further impair oxidative metabolism. The interruption of the TCA cycle decreases levels of malate and generates accumulation of some oncometabolites, such as fumarate, succinate, or α-KG. Under aerobic glycolysis, glutamine metabolism is enhanced, therefore contributing to α-KG accumulation as well. Now, the fermentative route provides less energy to the cell, obtaining the sum of 2 molecules of ATP per molecule of glucose in the glycolytic steps and a reduced amount of ATP molecules on this alternative and reduced oxidative phosphorylation. Green arrows indicate a net increase whereas red arrows show significant decreases. The figure was partly generated using Servier Medical Art, provided by Servier, licensed under a Creative Commons Attribution 3.0 unported license.

Almost one hundred years ago, in the decade of 1920, Otto Warburg for the first time proposed that cancer cells undergo a metabolic reprogramming that consists of two major features: (i) an increased glucose uptake and (ii) a metabolic switch to fermentative degradation of glucose incrementing lactate production (Figure 1). It is important to note that cancer cells present this special metabolic phenotype even when oxygen is available and that this type of metabolism is more inefficient when compared with the oxidative metabolism used by normal cells (Figure 1). Therefore, this phenomenon was dubbed aerobic glycolysis or the Warburg effect [12,13].

Despite being a major finding, this information remained mostly unnoticed until a few decades ago, when molecular biologists’ studies associated some oncogenes with cancer cell metabolism, and the Warburg effect was put back in the spotlight for cancer scientists. According to Warburg’s initial hypothesis, cancer cells engaged in this differential metabolism because they were damaged and their mitochondrial respiration machinery was perturbed [14]. Warburg went as far as to argue that the cause of cancer is this “irreversible injuring in cell respiration” [14]. However, it is now clear that mitochondrial function is essential for cancer cell viability, because elimination of cancer cell mitochondrial DNA (mtDNA) reduces their growth rate and tumorigenicity [15]. Different studies over the past decades have demonstrated that human tumors also respire; in fact, oxidative metabolism persists in several types of cancer and may even exceed the respiration rate of adjacent non-malignant tissue [16,17,18], everything depends on the type of tumor. For example, Bartman et al. [19] found that TCA cycle flux is suppressed in five primary solid tumor models; including spontaneous pancreatic adenocarcinoma, syngeneic pancreatic adenocarcinoma allograft tumors, spontaneous lung adenocarcinoma, xenograft colorectal cancer tumors, and flank Non Small-Cell Lung Cancer allograft tumors; but it is increased in lung metastases of breast cancer relative to primary orthotopic tumors. Furthermore, some recent data suggest that the observed mitochondrial rewiring that occurs due to the Warburg effect in some cancer cells, but not in all of them, is the result of an adaptive function [20,21]. In this regard, some studies suggest lactic fermentation might not be compensating mitochondrial deficiencies, but it could be an alternative mechanism to provide fuel for ATP production and to maintain large pools of glycolytic metabolites to support anabolic metabolism [21,22]. Still, now, the reason why a more inefficient metabolism is selected by some cancer cells remains intriguing.

It is well known that normal cells grow and regulate their metabolism according to different growth signals and receptors. In cancer, cells proliferate in a growth signal-independent manner with an accelerated biomass production that greatly increases the cellular anabolic demand while providing extra by-products that might contribute to tumorigenesis. Apparently, these accumulated biochemical intermediates are needed to maintain the altered proliferative rhythm, thus generating a dependency on this less-efficient metabolism [23,24,25]. Cancer cell’s dependency on the Warburg effect might also end up inducing autophagy or apoptosis, as the nutrient supply needs to be quite high to maintain the increased needs of the cell through aerobic glycolysis. Overall, it may seem that choosing the Warburg effect over other more efficient metabolisms is not a smart choice for cancer cells. However, other factors need to be taken into account. Although ATP production efficiency is radically reduced when compared to oxidative phosphorylation, this is not usually a problem for cancer cells, as resources tend to be unlimited, at least in the early phases of the disease [26,27]. Moreover, cancer cell requirements, other than ATP production, might be better satisfied with this alternative metabolism. For instance, glycolytic ATP generation takes place at a much higher velocity than through oxidative phosphorylation, so the ratio ATP generation/time is increased with this metabolic reprogramming [28,29]. Another reason could be that glucose and glycolytic derivatives serve as precursors for fatty acids, non-essential amino acids, and nucleotide synthesis [30]. In addition, the products released by glycolytic cells such as lactate can serve as substrates for mitochondrial oxidative phosphorylation in neighboring cancer or immune cells [31].

However, aerobic glycolysis is not the only change observed in tumor metabolism. In addition to glucose, proliferating cancer cells also rely on glutamine as a major source of biomass synthesis and as a source of energy, with glutamine feeding into the TCA cycle [32], a process also known as glutamine addiction [33]. Glutamine is the most abundant non-essential amino acid in the bloodstream that can be supplied from blood or synthesized by glutamine synthetase (GS) to meet the increased metabolic needs of rapid proliferating cells at a concentration as high as 0.5 mM [34,35]. Glutamine is further metabolized with ammonia into glutamate by glutaminases (GLS1/2) [36]. Then, it typically refills the TCA cycle with α-ketoglutarate (α-KG) through the glutamine–glutamate–αKG pathway, catalyzed by GLS and glutamate dehydrogenase (GDH) (Figure 1), and finally producing GTP/ATP, NADPH, and pyruvate. This pathway is called glutaminolysis [37]. Reflecting the highly heterogeneous nature of cancer metabolism, the degree of glutamine dependence differs among cancers and there are certain cancer types more prone to glutamine addiction than others, such as glioblastoma (GBM), pancreatic, breast or lung cancer, among others [38,39,40]. Despite being a non-essential amino acid, glutamine is necessary for the survival of the cancer cells that are addicted to it [32] and that is probably the reason why cancer can cause significant changes in the way glutamine circulates in the body, significantly enhancing the release of glutamine into circulation by skeletal muscle via upregulation of GS [41]. Furthermore, there are data suggesting that increased glutamine metabolism in cancer cells also promotes tumorigenesis and cancer cell survival by maintaining redox homeostasis and ATP supply [32,33]. Regarding the glutamine addiction process in GBM cells, some studies suggest that lactate is formed as a carbon residue through glutamine-derived malate [20,21]. The same research group described the fact that, on this same pathway, alanine is also produced as a way to get rid of excessive nitrogen groups. This process takes place through alanine aminotransferase (ALT) or GDH action [30,40,42]. Alanine formed this way can be secreted into the medium where it can accumulate and may serve as additional fuel for other cancer cells [43,44].

Considering all these data, it seems obvious that cancer cells obtain several benefits from altering their metabolism, including the Warburg metabolism, thus suggesting an adaptive function, although this is probably not the only reason behind the metabolic switch observed in cancer.

### 2.2. What Are the Causes of the Warburg Effect?

Although cancer cells obtain some benefits from the Warburg effect, the exact reasons why cancer cells switch toward it remain unclear, as well as the timing of this switch. Though understanding the full exact mechanisms behind the Warburg effect is still a great challenge, some of them have already been identified and studied.

It is true that the mutagenic status and/or altered pathways, both characteristic of cancer cells, are crucial for the regulation of the Warburg metabolism. However, the high cellular heterogeneity present in tumors has made it difficult to characterize the effects of specific alterations and their role in the origin of the Warburg metabolism switch. For example, strong evidence exists that some mutations in TCA cycle enzymes, such as succinate dehydrogenase (SDH) and fumarate hydratase (FH), are associated to and impaired oxidative phosphorylation that highly contributes to the metabolic reprogramming observed in cancer cells [45,46,47,48,49], as well as with an increased hypoxia-inducible factor (HIF-1α)-mediated glucose uptake and glycolysis [50,51,52]. These mutations that take place in mitochondrial genes have been addressed as responsible for the Warburg effect’s features [53]. These mutations that have been described in various cancer types are responsible for altering mitochondrial metabolism, which in turn enhances tumorigenesis and permits cancer cell adaptation to challenging environments, such as the hypoxic ones [35,54,55,56,57,58]. This alteration in mitochondrial functions is what led Warburg to state that a mitochondrial dysfunction was observed in cancer cells [14]. However, it is now globally accepted that having functional mitochondria is essential for cancer cells [57], and the Warburg effect is now considered as an altered or hardwired mitochondrial metabolism due to metabolic reprogramming and not a malfunction of the mitochondria.

However, even tumors that have hijacked their metabolism to suppress pyruvate oxidation and produce lactate use residual or reprogrammed aspects of respiratory mitochondrial machinery and can rewire their mitochondrial metabolism to maintain pools of TCA-cycle metabolites that can be further used as intermediates for anabolism and to generate oncometabolites [21,59,60,61].

For some time, it was thought that overexpression of HIF-1α due to transient hypoxic conditions partially induced the Warburg effect. This theory was based on evidence showing a HIF-mediated increased glycolysis in tumors, where glucose transporters and glycolytic pathway enzymes where upregulated [62]. HIF-1α is the main sensor and regulator of the hypoxia response in the cell, as it is degraded in the presence of oxygen, whereas it accumulates and triggers hypoxia-induced signaling under low oxygen concentrations. HIF-mediated hypoxia response is similar to the Warburg effect´s one, as metabolic conditions and cell requirements are similar. That is why HIF is very important in cancer fermentative metabolism as a key regulator of the process, even in the presence of oxygen, and that is why this process is called pseudohypoxia. Interestingly, lactate dehydrogenase A (LDHA) and pyruvate dehydrogenase kinase 1 (PDK1) expressions are increased by HIF upregulation, thus inducing an accumulation of lactate and reducing the supply of pyruvate to the mitochondrion [63]. At the same time that HIF provokes a decrease in mitochondrial respiration, it also optimizes the efficiency of glycolysis by regulating cytochrome C oxidase isoform ratios [64]. Nevertheless, some studies identified the Warburg effect´s onset prior to hypoxia on several tumors [65,66]. Therefore, it seems that HIF regulation of the Warburg effect takes place after the latter has already been activated by oncogenic pathways, such as phosphatidylinositol-3 kinase (PI3K) or Mitogen-Activated Protein Kinase (MAPK), and also by stabilization due to oncometabolite-induced prolyl hydroxilase domain (PHD) protein inactivation [67].

As we stated in the beginning of this section, just as with all other cancer hallmarks, the Warburg effect’s cause lays, ultimately, in gene mutations. For instance, the highly cancer-related pathway PI3K-Akt-mTOR induces an enhanced glucose capture, modulating GLUT1 expression and activating phosphofructokinase (PFK) and hexokinase (HK), among other enzymes, which contribute to high availability of glucose for the tumor cells, thus feeding the high needs of the Warburg metabolism [30]. Furthermore, HIF-mediated miR-199a5p inhibition leads to HK2 modulation and further increases on glucose availability [68]. Moreover, cancer cells express dimeric pyruvate kinase M2 isoform (PKM2) and upregulate PDK1, both of which inhibit pyruvate dehydrogenase (PDH), block pyruvate entrance into the mitochondrion to feed the TCA cycle, and result in an increased lactate production [69,70]. An increased AMP-activated protein kinase (AMPK) or c-MYC signaling, very common in cancer cells, also enhance the glycolytic flux through GLUT isoforms regulation or by inducing PFK or HK overexpression. The overexpression of glycolytic-involved genes (including LDHA, HK, GLUT1 or PKM2) has been shown in 24 types of cancer, including more than 70% of the total amount of human cancer cases [71,72,73]. At the epigenetic level, histone deacetylase (HDAC) inhibition has been shown to induce GLUT overexpression [74], and some sirtuins have been associated to Warburg’s metabolic switch through chromatin state regulation [75].

As previously mentioned, mutations in mitochondrial enzymes also play an important role in Warburg’s phenotype acquisition. Some examples include mutations in SDH, isocitrate dehydrogenase (IDH), and FH. What characterizes the Warburg effect is the lack of proportion between glycolysis and cellular respiration. Tumor cells that have switched towards a Warburg phenotype uptake high amounts of glucose in a rapid way and convert them into lactate even in the presence of oxygen. The proposed reasons to explain the aerobic glycolysis phenomenon are (1) that the glycolytic rate exceeds by far the maximal rate of mitochondrial pyruvate oxidation so lactate accumulation and secretion is unavoidable when glucose is so abundant [76]; (2) the overexpression of LDHA [77,78]; and (3) that due to mutations, the expression of some of the main TCA cycle enzymes is significantly decreased, thus reducing the entry of pyruvate into the TCA cycle and causing an impaired oxidative phosphorylation [45]. Ultimately, these mutations and the loss of oxidative metabolism contribute to oncometabolite accumulation (mainly succinate, fumarate, and α-KG), which is another important characteristic of aerobic glycolysis [67].

All these factors, and some others, such as the influence of tumor microenvironment (TME) components, the resulting acidosis, or mtDNA mutations, contribute to the metabolic switch [79,80,81]. However, this switch is not fully understood yet; there are gaps in the current knowledge of what initial changes lead to the Warburg status in cancer cells, mainly due to the high mutagenic variability observed in the cancer cells. Considering all this, the Warburg metabolism initiation needs further investigation that may lead to the development of new preventive or therapeutic strategies against cancer.

### 2.3. Main Features of the Warburg Effect

Although the reasons behind the switch from an oxidative metabolism to an aerobic glycolysis remain partially obscure, the Warburg effect has been extensively studied by now and its main characteristics and features have already been described.

As mentioned above, one of the main Warburg effect hallmarks is glucose uptake enhancement, mainly mediated by the increase in GLUT expression, meaning cancer cell survival also depends on the medium contents in nutrients capable to enter TCA cycle (that is why having alternative sources of carbon to enter the TCA cycle such as glutaminolysis-derived α-KG is so important for cancer cells). This upregulated acquisition is accompanied by an increase in glycolytic enzymes and a consequent increased glycolytic rate [77]. Moreover, pyruvate is not mainly metabolized to acetyl-CoA, as usual, but is diverted into lactate production, which is one of the main products responsible for driving the Warburg-associated increase in malignancy potential. As stated above, the upregulation of LDHA and PDK1, or mutations in these genes, are very important to induce the fermentative pathway [77]. Furthermore, glutaminolysis also contributes to lactate accumulation, which is responsible for acidification of the TME, which is important for tumor malignancy. This acidification of the medium happens because the cell tries to alleviate lactate accumulation in the cytoplasm through upregulation of the monocarboxylate transporter (MCT-4) (Figure 1), which ejects lactate from the cell, a process that is also regulated by HIF-1α [82]. However, these transporters work as symporters and, at the same time, they eliminate lactate and eject protons into the extracellular space, generating its acidification [83,84]. Lactate externalization has two more consequences: the first one is that extracellular lactate is internalized by other cancer or tumor microenvironment (TME) cells that express MCT-1. Then, those cells use the excess of lactate to trigger mitochondrial oxidative metabolism, generating a process known as the reverse Warburg effect, becoming more efficient in energy production [85]. The second consequence is that lactate can bind to G protein-coupled receptor 81 (GPR81) which, in turn, activates certain pathways such as PI3K/Akt, favoring cancer progression [86].

Another Warburg effect hallmark, and a general cancer trait, is the enhancement of anaplerotic reactions. The accelerated metabolism and the increased use of some molecular component sources make it necessary to induce additional anabolic pathways in order to regenerate Krebs cycle intermediates. Some of the enzymes relevant for these pathways are pyruvate carboxylase, aspartate aminotransferase (which restores oxaloacetate levels which are important for citrate synthesis), propionyl-CoA carboxylase (which functions in odd-chain fatty acid utilization in order to generate succinyl-CoA), or glutaminase (which intervenes in glutamine catabolism, generating α-KG) [87].

All these changes in metabolism not only allow cancer cells to obtain more energy, but are also linked with the upregulation of protumoral signaling pathways, helping cancer cells to gain malignancy. This is another reason why the Warburg effect might constitute a selective advantage for cancer cells; this metabolism enhances the availability and function of certain metabolites that promote cancer progression, and that is why they are called oncometabolites. These molecules usually have a specific role in metabolism, but their excessive accumulation under Warburg conditions help cancer progress. The best known oncometabolite is lactate, whose pivotal role in cancer progression has been already summed up in an extensive review [82]. Other important oncometabolites include 2-hydroxyglutarate, fumarate, and succinate (Figure 1) [88]. However, the role of other TCA-cycle metabolites in cancer initiation and progression should not be disregarded, even if they are not considered oncometabolites, and investigating them may open new and interesting topics in the research against cancer. A detailed review about this can be found in the work of Eniafe and Jiang [89].

The hexosamine biosynthetic pathway (HBP) generates UDP-GlcNAc, another oncometabolite. This pathway is also upregulated in cancer under the Warburg effect and both UDP-GlcNAc, its final product, and some intermediates are able to modulate signaling pathways that favor tumor progression. The HBP works as a metabolic sensor, so when more nutrients are present, both glycolytic and HBP pathways are enhanced [90].

Another relevant feature that occurs in the Warburg metabolism concerns reactive oxygen species (ROS). The role of ROS in cancer has been studied for many years, and it is widely known that cancer cells have a higher production of ROS. In addition, when metabolism is enhanced, especially in those cells that still maintain oxidative phosphorylation, ROS production rises further. To deal with this oxidative stress, the pentose phosphate pathway (PPP) uses glycolytic intermediates, which are hijacked from glycolysis, to generate NADPH as a protection against the increased amount of tumor-generated ROS [91].

Some of the previously described features, as well as other characteristics, have been useful to understand not only cancer metabolism, but also cancer behavior in general, which can ultimately impact clinical cancer management. For instance, based on the increased glucose consumption rates by cancer cells, Fluorodeoxyglucose (FdG) is commonly used for positron emission tomography (PET) imaging, which allows to diagnose and follow up tumors [62,92].

### 2.4. Classical Approaches Targeting the Warburg Effect to Fight Cancer

Characterizing cancer metabolism is not only important to cancer diagnosis, but it may also help in cancer therapy development by targeting specific metabolic cancer cell features. This is why having a better understanding of Warburg metabolism and its main characteristics, such as accumulated oncometabolites, is so important, as it may represent a new avenue of research to discover new anti-cancer strategies.

In an attempt to impair cancer metabolism by targeting the Warburg effect characteristics, some treatments have already been tested. Some approaches in this line include the inhibition of glycolytic enzymes; for instance, HK2 inhibitors, such as lonidamine, have reached clinical trials in combination with other antitumoral compounds, although no significant effects were obtained or toxicity appeared [93]. 3-bromopyruvate is another HK2 inhibitor that has shown good preclinical results in liver and prostate cancer [18,94]. The use of GLUT inhibitors is also a common approach. For instance, WZB117 increased the effect of other treatments, while STF-31 showed positive preclinical results [95,96,97]. Another way to inhibit the Warburg effect is targeting lactate production by LDHA-mediated approaches. Some examples are urotilin M6 and FX11, which have been proven to inhibit LDHA in preclinical studies on hepatocytes and adipocytes, and on 15 patient-derived TP53-mutated mouse xenografts, respectively [18,97,98,99,100]. MCT inhibition is also an interesting way to attack cancer metabolism, and AZD-3965 is a good example of an MCT1 inhibitor that is currently in clinical phase of research for advanced cancers [97,101,102]. Many HIF inhibitors have also attracted great interest in anti-cancer therapy research. Among these, two synthetic molecules, PT2385 and PT2399, have shown good results in renal carcinoma cell lines [18,103]. Other attempts include pyruvate kinase M2 inhibition [104] or ketogenic diets [105]. It is true that in the past decades have been some controversy about the beneficial role of ketogenic diet in human health [106,107,108], however, in the specific case of cancer this type of diet targets the Warburg effect in tumor cells [109]. The characteristics of ketogenic diet, a high-fat, low-carbohydrate diet with adequate amounts of protein, creates an unfavorable metabolic environment for cancer cells by drastically reducing the amount of glucose available for tumor cells [109]. Furthermore, in response to an increase of ketone bodies under ketogenic diet feeding, CD4+ and CD8+ T cells are metabolically reprogrammed to rely on OXPHOS and they are able to secrete more cytokines [110]. In addition, it appears to sensitize most cancers to standard treatment by exploiting the reprogramed metabolism of cancer cells and by boosting effector T cell functions, making this diet a promising candidate as an adjuvant cancer therapy [105,110].

Some possible molecules to target glutamine metabolism have also been developed. One candidate is CB-839, under the commercial name Telaglenastat, which has proven to sensitize cancer cells to radiation in cervical cancer and in head and neck squamous cell carcinoma, in in vivo models, and has even reached clinical trials against some tumors. Other approaches are L-asparaginase (L-ASP), which induces glutamine depletion and increases radiosensitivity and induces cell cycle arrest in ARCaPM cells, or JHU083 and DRP-104, which are general glutamine inhibitors [98,102,111,112].

Another good option is targeting the Warburg phenotype by tackling more upstream actors in the metabolic pathway. For example, sirtuin 6 (SIRT6) is a tumor suppressor that inhibits HIF-1α and, as a consequence, reduces HK, LDHA and GLUT1 [75,113].

Recently, the different complexes of the ETC have attracted interest as a possible target to develop new therapies to compensate or reverse the mitochondrial switch observed in cancer [26,114,115]. In this regard, the work by Moreira et al. [116] not only summarizes the fundamental role of cytochrome c oxidase, complex IV of the ETC, in the effectiveness or not of chemotherapy, immunotherapy and probably radiotherapy treatments; but it also provides new interesting anti-cancer metabolic therapy strategies to hijack Warburg phenotype. They proposed that photosensitizers such as methylene blue, chlorophyll, and protoporphyrin could play an intermediary role in the electron decongestion of ETC by catalyzing the activation of ^3^O_2_ into ^1^O_2_ and thus promoting apoptosis by accumulation of ROS species in tumoral cells, especially those that are resistant to conventional treatments [116].

Unfortunately, despite the great effort made in the last decade to target cancer metabolism, the clinical applications are still modest. Cancer cell plasticity is enormous, and that enables them to compensate the lack of a specific pathway by obtaining energy through other means. For example, glycolysis inhibition can be compensated by glutaminolysis [117,118], as glutamine drives the glucose-independent TCA cycle, as described above.

So far, it has been difficult to obtain enough specificity without reaching high levels of toxicity with the previously explained options [119]. A potential alternative for these problems is the use of combined therapies to avoid resistance, for instance, by simultaneously inhibiting glycolysis and glutaminolysis. Another way would be finding new methods to deliver the drug specifically on the tumor site to avoid toxicity in normal tissues and perhaps increase the local dosage.

Another problem for these therapeutic options is that not all tumor cells rely on aerobic glycolysis; for instance, cancer stem cells and other slow-proliferating cells are very resistant to these treatments because of their lower growth rates and different metabolism. That is probably the reason why very few Warburg-based treatments have reached clinical phases [18]. However, targeting the Warburg effect or its main features, including its associated oncometabolites, is still a promising alternative for anti-cancer therapy research. More investigation is needed to properly address the challenges of this still mostly unexplored route.

## 3. Succinate, the Forgotten Oncometabolite?

Focusing on specific metabolites related to the Warburg effect may be a good option inside the different alternatives to target Warburg metabolism in the fight against cancer. As previously mentioned, one of those metabolites that plays a major role in cancer development is succinate.

### 3.1. Succinate Metabolism and Accumulation

Succinate is the anionic and main form of succinic acid. Its major role lays on metabolic function, which is part of the Krebs cycle. It is synthetized by succinyl Coenzyme A synthetase from succinyl CoA, which separates the CoA moiety generating either an ATP or GTP molecule in the process. Succinyl CoA is mainly synthetized from α-KG during the TCA cycle, so the main carbon sources contributing to succinate formation are glucose used in the Krebs cycle and glutamine used through glutaminolysis [37,120] as previously explained in this review. Moreover, succinate metabolic reactions are associated to some other pathways such as branched-chain amino acids synthesis, the heme group synthesis, the gamma-aminobutyric acid (GABA) shunt, or ketone bodies usage [120,121]. Succinate degradation is accomplished by SDH, which converts FAD into FADH2 and generates fumarate. SDH is part of complex II of the mitochondrial ETC. SDH oxidizes succinate, reducing ubiquinone with the resulting electrons, which can also contribute to the electrochemical gradient on the mitochondrial inner membrane to generate ATP [121,122]. Furthermore, SDH has an important role because one of the main causes for succinate accumulation in the cell is SDH inhibition.

Succinate accumulation is relevant because it is a main feature observed in cancer [123,124]. As just mentioned, SDH inhibition or dysfunction is one of the main causes for this accumulation [125]. Some SDH inhibitors include tumoral necrosis factor receptor-associated protein 1 (TRAP-1) and arrestin beta 1 (ARRB1) [121,122]. A deficiency of SDH activity can also be explained by germline mutations in its subunits (SDHB and SDHD) that result in a collapse of the SDH catalytic activity, leading to succinate accumulation and excessive ROS generation [126]. In addition to SDH subunit mutations, the loss of SDH function may be the result of loss of heterozygosity [127] or reduction in the SDH subunits’ expression in diverse cancers [128,129]. Although the mechanism by which the SDH expression is reduced in cancer cells is not entirely clear, recent results suggest that a reason could be the degradation of SDH transcripts mediated by microRNA-210 upregulation [128]. Other causes for succinate accumulation include a massive production of succinate by the immune system as part of the inflammatory response, an excess of GABA or glutamine [121,130], or mutations in other enzymes of the TCA cycle as IDH and FH [131]. Interestingly, succinate accumulation induces overexpression of its own receptor in some tissues [121,130]. These genetic alterations that lead to succinate accumulation have been widely described in cancer cells where a metabolic reprogramming towards a Warburg phenotype is also observed. Many works, including Warburg’s original work, propose that these mutations are one of the causes of the metabolic rewiring observed in tumors. This could be interpreted as succinate accumulation happening before or at the same time as the Warburg effect instead of as a result of the metabolic reprogramming. However, other works point out that the alterations observed in the TCA cycle under the Warburg effect may also be the main source of succinate accumulation [132]. Measurements of ^(13)^C-labeled metabolites in [3-(13)C]aspartate-treated cells showed that anaerobic pathways of αKG/ASP metabolism, in order to generate ATP, yielded succinate as an end product [132]. Furthermore, it is known that when a cell switches to fermentative metabolism, the Krebs cycle is abruptly interrupted rather than progressively suppressed. This interruption takes place at specific checkpoints, mainly on the conversion of citrate to α-KG and on the succinate to fumarate reaction. Therefore, the Warburg-induced Krebs’s cycle shut-down leads to succinate and citrate increases [133,134,135,136]. Lastly, increased concentrations of succinate have also been described because of HIF-independent hypoxic mechanisms, perhaps because of the reversion of the SDH reaction. This lack of SDH function has also been described under ischemic conditions, in non-tumoral cells [137]. In fact, succinate concentration under normoxic conditions at the mitochondrial matrix was found to be 0.1 mM, whereas under hypoxia, it arose to 6.0 mM [121]. Also related to the hypoxic environment, typical for tumors, several studies show a succinate increase after ischemia in rat models [138,139]. Moreover, high lactate levels contribute to succinate accumulation [140], maybe because ischemia reduces SDH function [137], inducing succinate accumulation, and lactate (and some other oncometabolites) can trigger pseudohypoxia, which activates a mechanistic pathway comparable to the ischemic one [65,120,136]. Considering all these data, it seems that there are different features that lead to succinate accumulation; some of them may happen before the metabolic reprogramming of the tumoral cell as a consequence of the impairments in the oxidative metabolism, and some can happen due to the stimulation of aerobic glycolysis in cancer cells. What cannot be denied is that succinate accumulates in the cancer cells that exhibit a Warburg phenotype and that the Warburg metabolism and succinate are linked, both contributing to each other.

### 3.2. Succinate Role in Cancer

**Figure 2:**
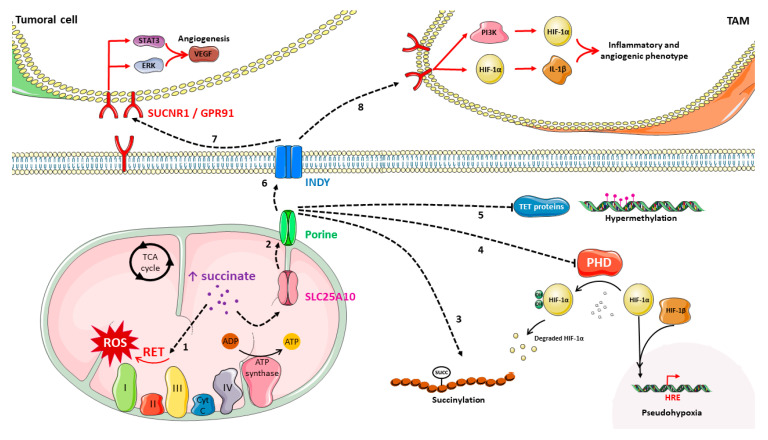
Effects of succinate accumulation related to cancer: Fumarate excess, caused by the accumulation of succinate, induces a complex III saturation, which generates an electron flow reversion towards complex I—Reverse Electron Transport (RET)—where reactive oxygen species (ROS) are generated (1). Accumulated succinate inside the mitochondrion can escape to the cytoplasm by crossing the inner mitochondrial membrane through Solute Carrier Family 25 Member (SLC25A10) and the outer membrane through porines (2). Succinate is needed for succinylation, a post-translational modification on lysine residues (3). Succinate is responsible for Prolyl Hydroxylase Domain (PHD) protein inhibition, inducing Hypoxia-Inducible Factor (HIF-1α) stabilization and the consequent transcription of HIF target genes under normoxic conditions (4). Succinate inhibits Ten-Eleven Translocation (TET) enzymes, which are histone demethylases inducing a hypermethylated status (5). Succinate can be transported outside the cell, probably through the I’m Not Dead Yet (INDY) transporter (6). Succinate can bind to Succinate Receptor 1 (SUCNR1) on neighboring cancer cells and induce Vascular Endothelial Growth Factor (VEGF) expression through Extracellular Signal-Regulated Kinases (ERK) or Signal Transducer and Activator of Transcription 3 (STAT3) activation, inducing angiogenesis (7). Succinate can bind to SUCNR1 on Tumor-Associated Macrophages (TAM), triggering the phosphatidylinositol-3 kinase (PI3K)/HIF signaling or the HIF-dependent, Interleukin-1 β (IL-1β) activation. These pathways induce a TAM angiogenic and inflammatory phenotype (8). The figure was partly generated using Servier Medical Art, provided by Servier, licensed under a Creative Commons Attribution 3.0 unported license.

It is important to remember that succinate is also an oncometabolite, so its functions are not limited to mitochondrial metabolism. Succinate is an intermediate normally confined to the mitochondrial matrix. However, under exceptional circumstances such as cancer, succinate can accumulate in the matrix, further leaking into the cytoplasm [127]. Succinate can reach the cytoplasm by crossing the inner mitochondrial membrane through dicarboxylated transporters such as Solute Carrier Family 25 Member 10 (SLC25A10), which is a succinate–fumarate/malate transporter, and the outer mitochondrial membrane through porines [141]. In fact, succinate can even be secreted, and it can function at the extracellular space. The mechanism by which succinate is externalized seems to be tissue-specific and not yet fully understood. However, a good candidate is the I’m Not Dead Yet (INDY) transporter, which is an anion independent sodium interchanger that might be responsible for succinate transport into the bloodstream. The fact that succinate can exert different functions outside the mitochondrion is very relevant for cancer development and progression (Figure 2) [141] and, which is even more important, it seems that intracellular and extracellular increased succinate exerts different pro-tumoral activities [127].

A major role of succinate lays in metabolism as part of the Krebs cycle, but its Krebs cycle-independent functions are exacerbated in cancer. Extracellular succinate also has a hormone-like behavior when binding to its membrane receptor called G-protein coupled receptor 91 (GPR91)/succinate receptor 1 (SUCNR1). For example, extracellular succinate binds to SUCNR1 and induces angiogenesis through signal transducer and activator of transcription 3 (STAT3) and activation of extracellular signal-regulated kinases (ERK1/2). This pathway induces vascular endothelial growth factor (VEGF) upregulation [124,130]. SUCNR1 also induces HIF stabilization, which generates Interleukin-1 β (IL-1β) overexpression, which, in turn, activates the M1 phenotype in macrophages that triggers tumoral angiogenesis (Figure 2) [134,142]. Succinate´s favorable role to cancer progression seems clear and, actually, SUCNR1 overexpression has been identified in different tumor types, such as renal cancer [143].

Another way through which succinate can have a protumoral role is by participating in mitochondrial ROS production, since SDH is very important in ETC. When succinate accumulates in the cell, massive amounts of fumarate are produced, and this can induce reverse electron transport (RET). Under these conditions, complex III reaches saturation and electrons invert their flow through the ETC and reach complex I, which generates ROS to get rid of those electrons (Figure 2) [121,130].

Succinate procancer role is also associated to epigenetics as intracellular accumulated succinate inhibits the 2-oxoglutarate-dependent dioxygenase (2-OGDD) family. Some of these proteins are histone and DNA demethylases. Moreover, both succinate and fumarate inhibit a family of Ten-Eleven Translocation methylcytosine dioxygenases (TET) and Jmc family histone lysine-specific demethylases (KMD). Therefore, high levels of succinate can induce a DNA hypermethylated status and genetic silencing, thus affecting tumor suppressor genes (Figure 2) [88,144].

Other 2-OGDD inhibited by intracellular succinate are PHD proteins. This inhibition triggers a pseudohypoxic state where, even in the presence of oxygen, HIF stabilization and accumulation takes place, inducing transcription of HIF’s target genes, many of which have protumoral effects (Figure 2) [145]. Collagen prolyl-4-hydroxylases are also inhibited by succinate, which also induce HIF stabilization and epithelium-mesenchymal transition (EMT) [146]. Other studies have described succinate´s role in inflammatory processes, and it is well known that inflammatory states maintained over time also contribute to cancer progression [130,147].

Succinylation is a posttranslational modification of proteins that requires succinate. The way in which this process happens in the cytoplasm and the identity of the succinylation moiety are not yet well known, but the presence of a succinyl group on a protein has been demonstrated to be important in some types of cancer (Figure 2) [144].

The increased levels of succinate in cancer cells are a fact, and, which is more important, this elevation is not limited to the tumor cells or microenvironment. Elevated serum succinate levels can be found in some cancer patients, suggesting that cancer cells secrete succinate into the circulating blood, making it a potential candidate for a cancer biomarker [123,148]. However, the implications of this increased succinate in the whole body are not fully understood yet and more research is needed in this aspect.

Even though the succinate increase has not been described as a main Warburg feature (only increased glucose uptake and lactate secretion have been), as previously mentioned, TCA cycle abrupt interruption, specific Warburg-leading mutations, and the increased anaplerotic shunt or HIF stabilization lead to succinate accumulation in a Warburg context. This is supported by the fact that increased lactate, typical of aerobic glycolysis, is accompanied by increased succinate levels [140]. Moreover, cancer cells with fermentative metabolism secrete succinate and activate the M1 phenotype in surrounding macrophages, which in turn promotes angiogenesis induction and tumor progression [123,134].

In the context of Warburg metabolic switch that occurs in cancer cells, succinate accumulation is therefore expected. According to all the protumoral effects related to succinate already listed in this review, it is logical to think of succinate as an oncometabolite that can be targeted to find new therapeutic strategies against cancer.

### 3.3. Succinate: A New Alternative to Target Cancer Metabolism?

As previously mentioned, several approaches to target cancer metabolism have been attempted. However, despite the great effort made, little advances have been reached in the field. Therefore, taking into consideration the numerous links between enhanced succinate levels and tumor progression in a Warburg context, we propose succinate-directed targeting as a possible alternative for cancer treatment. However, one ambiguous point in this approach is the fact that when inhibiting succinate production, there is not only an interruption of the oncometabolite functions exacerbated under Warburg’s metabolism, but also an inhibition of the TCA, thus decreasing ROS generation. The role of ROS in cancer is a double-edge sword; on the one side, high levels of ROS favor chemotherapeutic or radiotherapeutic success and have tumoral suppressor actions; for instance, triggering apoptosis or inhibiting the cell cycle. On the other side, ROS can also activate protumoral pathways and induce DNA mutations [149,150]. This does not mean that succinate is not a good candidate for anti-cancer therapy development, but researchers need to be more careful in regard to ways to approach these investigations.

Among the different possible ways to use succinate as a potential way to control cancer growth and metastasis, three are the most promising.

#### 3.3.1. SUCNR1 Could Be the Key to Suppress Succinate Extracellular Protumoral Actions

It is known that SUCNR1 serves as an extracellular receptor for succinate. Several studies have described that succinate-activated SUCNR1 transmits signals via non-canonical signaling pathways such as PI3K that selectively promotes cancer cell migration or metastasis [123,127]. Succinate-SUCNR1 activation triggers angiogenesis [151] and activates TAMs, thus SUCNR1 blockage or signaling interference could be of great help.

As happened with succinate, SUCNR1 canonical activation pathways are part of the inflammatory response, thermogenesis, skeletal muscle adaptation to exercise, and the regulation of renin–angiotensin axis and hypertension [152,153,154,155,156,157]. That is why targeting a receptor involved in several different biological processes is a delicate approach, since side effects and general toxicity may appear. That is probably the reason why very few attempts have been made to inhibit succinate action by targeting its receptor. Still, there are examples in the literature; one of them is NF-56-EJ40, a SUCNR1 inhibitor that was proposed to block immune cell migration and inhibit succinate-induced gene regulation on M2 macrophages [158]. Bhuniya et al. [159] also found a small molecule that acted as a selective inhibitor of human SUCNR1, as demonstrated using a pharmacodynamics in vivo assay measuring succinate-induced increases in blood pressure. In another study, a SUCNR1 knockdown combined with chemotherapeutic treatment was tested in high-glutamine-expressing tumors. This combined therapy killed cancer cells since succinate accumulation induced large amounts of ROS, which were cytotoxic. Therefore, in this case, instead of reducing succinate levels, the opposite approach was used, taking advantage of excessive succinate accumulation [160].

Additional investigations are needed to clarify the potential side effects of these treatment strategies and the effect these inhibitors could have on normal cells and tissues to have a better understanding of their benefit–risk ratio and whether they could still be considered as a suitable option and a new alternative pathway in the research of new anti-cancer drug development.

#### 3.3.2. Sirtuin 3, a Booster to SDH Catalytic Activity

Although targeting SUCNR1 can block the extracellular effects of succinate accumulation, it does not have a major effect on the actions that increased succinate levels can have inside the mitochondria matrix and in the cytoplasm. That is why a more efficient alternative could be targeting SDH action in order to avoid succinate accumulation in the first place.

SDH overexpression was induced with positive anticancer effects comparable to rapamycin, naringin, sirtuin 3 (SIRT3), or nuclear respiratory factor 1 (nrf1) [125]. It was also shown that overexpressing SDH in ovarian cancer cells induced apoptosis and stopped cancer cell proliferation. This strategy also inhibited cancer cell migration and invasion, probably because of the concomitant reduction in HIF levels [129].

Among the molecules with potential to impact on SDH activity, SIRT3 is a good candidate. SIRT3 is a member of the sirtuin family that can be found in the mitochondrial matrix where it functions as a deacetylase [161]. The acetylation modifications that are regulated by SIRT3 are essential for maintaining mitochondrial function [162]. That is why it is not surprising that SIRT3 is involved in aging, neurodegeneration, liver disease, kidney disease, heart disease, and other metabolic diseases [162], and in cancer, too [161,163]. Moreover, SIRT3 expression is downregulated in high glycolytic and proliferative hepatocellular carcinoma cells of human patients, xenograft models, and cell lines [164], showing a correlation between this protein and cancer.

Among its different functions, SIRT3 regulates lysine acetylation of SDHA subunit. SIRT3 binds to SDHA and increases SDH electron transfer and catalytic activity, thus preventing succinate accumulation and favoring its degradation towards fumarate [165]. Thanks to its capacity to control succinate accumulation, to hinder cancer metabolic changes [166], and its role regulating the acetylation of several mitochondrial proteins including superoxide dismutase II, which in turn reduces the amount of ROS in the mitochondria [167], SIRT3 has been described as a tumor suppressor in glycolysis-dependent cancers [166,168].

However, its role in other types of cancer, where the oxidative phosphorylation is still the most important source of energy supply, needs to be clarified, as SIRT3 promotes oxidative phosphorylation and inhibits glycolysis [166], and some recent studies demonstrated that SIRT3 can exert a pro-tumoral role depending on the metabolic circumstances of the tumor [169,170,171].

This does not mean that SIRT3 is not a suitable candidate for anti-cancer therapy development, but its application may be limited or restricted to those tumors that have reprogrammed their mitochondrial machinery towards a “Warburg phenotype” and may benefit from a rewire of their metabolism. In fact, SIRT3 is already being studied as a therapeutic target for several diseases, including cancer, and a series of small-molecule and other regulatory compounds targeting SIRT3 have been discovered or designed synthetically. Compounds that act as positive modulators of SIRT3 by increasing its expression have already shown favorable therapeutic effects in other pathologies such as cardiac hypertrophy [172,173], acute kidney injury [174,175], osteoarthritis [176] or liver metabolic diseases [177].

Interestingly, most of SIRT3 positive modulators have a vegetal origin. For instance, one of the most potent and most studied substances that increases SIRT3 expression is Honokiol, a lignan isolated from the bark, seed cones, and leaves of trees belonging to the genus *Magnolia*. Honokiol-induced SIRT3 overexpression effects have been tested with positive results in heart disease [173,178], renal disease [179] and vitiligo [180]. In these studies, Honokiol could increase SIRT3 expression and deacetylation activity, thus improving mitochondrial rate of oxygen consumption and inhibiting ROS synthesis [173], improving ROS elimination [181], regulating the immune system response by inhibiting the NF-κB-TGF- β1/Smad via [179] and blocking the overexpression of the Akt pathway [173]. All of these actions could have a positive impact on suppressing tumor growth, too, so Honokiol could be a candidate in the development of new therapies against cancer. In fact, Pillai et al. [178] already tested a combined treatment with doxorubicin, a chemotherapeutic drug of choice for a wide variety of cancers, and Honokiol showed a protective action against doxorubicin-induced damage in cardiomiocytes without affecting the anti-cancer activity of the drug. This suggests that Honokiol could contribute as an adjuvant therapy of chemotherapy and it is safe to use. Furthermore, some studies are already testing Honokiol as an anti-cancer treatment by itself with promising results in in vitro and animal models [182,183,184]. However, not much has been considered in these studies about the impact of this treatment on different oncometabolites, so further research is needed to confirm whether the positive anti-tumoral effects observed after Honokiol treatment are partially mediated by succinate accumulation prevention or not, as this compound has an outstanding antioxidant capacity that can be in part explained by its structural characteristics based on polyphenols [185]. Silybin is another natural plant-derived SIRT3 activator that has also been tested in combination with a chemotherapy drug, cisplatin [174]. The results obtained by Li et al. suggest that silybin is a pharmacological activator of SIRT3 capable of protecting against cisplatin-induced tubular cell apoptosis by improving mitochondrial function [174]. Thus, silybin could also serve as a potential clinical protective adjuvant treatment in cancer chemotherapy.

However, natural SIRT3 activators are not the only ones that have been tested so far. Indeed, recent cancer studies have tried to improve cancer treatment results through SIRT3-enhanced expression. Jo et al. [164] discovered that PD0332991, a highly selective inhibitor of CDK4/CDK6 kinases with the ability to block phosphorylation activity of retinoblastoma (Rb) [186], can significantly enhance the expression of SIRT3 in hepatocellular carcinoma cells, thus improving the anti-cancer action of sorafenib [164], also known as Nexavar, an orally active multikinase inhibitor, which has been used as a first-line chemotherapeutic agent [187]. In another study where hepatocellular carcinoma cells were infected with lentivirus overexpressing SIRT3, it was shown that SIRT3 overexpression significantly enhanced cellular susceptibility to three chemotherapeutic agents [188]. A more recent study used ABT737, a compound that inhibits Bcl-2 [189], to activate SIRT3 expression in ovarian cancer, and the results showed that ABT737 enhanced the sensitivity of ovarian cancer cells to cisplatin due to the overexpression of SIRT3 and the regulation of mitochondrial fission [190].

Although targeting SIRT3 seems a promising new avenue in anti-cancer therapy development, more investigation is needed to ensure whether the already observed positive effects of SIRT3 positive modulators in fact occur due to a reduction in succinate levels, or if they rely on other mechanisms of action. Furthermore, the development of more specific SIRT3 activators would be desirable, as so far only less selective compounds with biological activity themselves have been tested, making it difficult to identify the exact role played by SIRT3 in the observed anti-cancer effects of these compounds. However, the benefits of using SIRT3 positive modulators as adjuvants in cancer treatment seem clear, and many patients could benefit from them. In this matter, it is important to reiterate that the available data so far show that only tumors with a glycolytic metabolic reprogramming may benefit from this kind of therapy, so a metabolic screening of the tumors prior to therapy selection would be the appropriate protocol if these therapies reach clinical practice.

#### 3.3.3. TRAP-1, an Inhibitor of SDH

As mentioned above, TRAP-1 inhibits SDH, thus reducing the TCA and the ETC. Therefore, SDH inhibition is one of the main features that induces succinate accumulation and the corresponding HIF-1α stabilization. In this context, the hypothesis that inhibiting or reducing TRAP-1 activity may have beneficial effects over cancer seems promising. Moreover, it has been suggested that TRAP-1-silenced cells lose their cancer-transforming potential, which can be further recovered with murine cDNA TRAP-1 transfection [191]. Surprisingly, another study that described the respiratory Complex 2 stabilization by TRAP-1 also showed that TRAP-1 knockdown caused SDH inhibition [192], which was contrary to what would have been expected.

Also related to cancer metabolism, TRAP-1 promotes aerobic glycolysis by decreasing oxidative phosphorylation and enhances glutamine metabolism [193,194]. However, some contradictory data comparing WT versus TRAP-1 K.O. mice hepatocytes shows contradictory results that need to be further analyzed [194].

In addition, TRAP-1 is a Myc oncogene target [195], and its overexpression was detected in several tumor cells resistant to treatments, probably because it protects cancer cells from oxidative apoptosis through cyclophilin D (CypD) antagonism [196,197]. In other studies, breast or prostate cancer cells did not have increased levels of TRAP-1, but it was important for their different stages of progression [198,199]. Furthermore, in the case of colorectal cancer, an overexpression of TRAP-1 was associated to a poor prognosis [200]. Considering all these data, it seems obvious that in most cases, TRAP-1 expression is higher in cancer cells than in normal tissues [201,202].

However, TRAP-1 is a chaperone from the heat shock protein 90 family (HSP-90), and its actions are not limited to the metabolic level, but they affect many cellular aspects such as mitochondrial homeostasis, as well [202,203]. Related to its mitochondrial homeostatic functions, TRAP-1 protects cells from apoptotic death and from oxidative stress [203,204,205]. It has implications in subcellular trafficking of Rb during mitosis [203]. TRAP-1 is also crucial in some other pathologies such as Parkinson disease, as a mutation in PTEN-induced Putative Kinase (PINK1), which phosphorylates and activates TRAP-1, is present in the disease, where PINK1-mutated cells cannot activate TRAP-1 and apoptosis occurs [206].

Some compounds have been already proven to inhibit mitochondrial HSPs and they could be useful to block the action of TRAP-1. Gamitrinibs, which are small molecules that inhibit HSPs, are specially designed to accumulate in mitochondria instead of the cytosol and selectively inhibit TRAP-1 in this organelle [207]. A preclinical model of prostate cancer showed good results in terms of toxicity and inhibitory activity [208,209,210]. Another similar strategy was already developed, consisting of an oxide nanoparticle bound to geldanamycin, which is a TRAP-1 inhibitor, and the mitochondria localization signal (MLS). Although the particle was stable and decreased cancer cells metabolism in vitro, it had no significant effect on cell viability [211]. Another general HSP90 inhibitor called NVP-HSP990 also presented antitumoral activity against different types of tumors in preclinical studies [212].

For all these reasons, the idea of using an HSP inhibition strategy as a possible therapy for succinate accumulation seems plausible. Despite the variety of functions that TRAP-1 displays, the specific abundant expression of this protein in most tumors, in contrast to the little expression in non-transformed cells, even in proliferative ones, opens the possibility to use the inhibition of TRAP-1 as a new cancer therapy, especially considering most of the functions it exerts are protumoral [191,209]. However, it is important to consider that the potential success on these approaches relies on achieving specific actions of these inhibitors limited to the mitochondria in order to reduce possible adverse effects caused by TRAP-1 inhibition in the cytoplasm.

In conclusion, TRAP-1 potential for cancer-directed therapy is really high, although succinate’s responsibility on TRAP-1 protumoral involvement needs to be further analyzed and cleared, as it may be less relevant than other TRAP-1 targets.

## 4. Conclusions

In conclusion, cancer metabolism research has allowed the scientists to advance in cancer biology knowledge collection, providing new bases for the development of better and more advanced anti-cancer treatments. In this line, the Warburg effect features have been summarized, and especially the importance of succinate accumulation that happens in a Warburg metabolic reprogramming has been analyzed. Evidence shows that succinate favors tumor progression. In fact, not only does it trigger cancer favoring signaling pathways at the cancer cell, but it also has protumoral effects on neighbor cancer cells and even other cells in the TME, such as immune system cells.

Targeting such a relevant piece in cell metabolism machinery constitutes an important challenge, but benefits from inhibiting succinate cancer-related functions might represent a good advantage for cancer patients. In this review, we have summarized the three most interesting and promising pathways to reduce succinate actions as an oncometabolite and/or prevent its accumulation by targeting its receptor, SUCN1, or enhancing its degradation by SDH via SIRT3 or TRAP-1. We have also presented evidence of different compounds that have already been tested with promising results in cancer and other diseases targeting these three possible strategies, as well as discussed the potential problems that may arise from their use. It is important to note that, so far, none of these approaches have reached clinical applications, and more research about the role of succinate in cancer is needed as the role of this oncometabolite is not absolutely clear in the observed beneficial effects of these therapies.

Among those three, probably the most studied so far and the one that has provided the best results are the new therapies focused on increasing SIRT3 expression. Different natural and chemical compounds have been tested so far with promising therapeutical results in other diseases, as explained above. However, these results should be carefully considered in cancer, as according to previous data, SIRT3 activation is beneficial only in glycolysis-addicted cancers that have switched towards a “Warburg phenotype”. In other tumors where oxidative phosphorylation is conserved and even enhanced, SIRT3 overexpression can have protumoral undesired effects. For instance, these new therapy options have provided the best results when combined with other chemotherapy agents. Therefore, even if they cannot be used alone, their potential as candidate adjuvants should be considered. These options should be investigated for TRAP-1 inhibitors, too, as this could open new avenues to improve the current available treatments for cancer patients.

However, the above-proposed therapy strategies are not the only ones that can be considered and tested. As previously mentioned, obtaining localized effects of treatments on tumors to avoid spread toxicity is another key point in the research of new anti-cancer therapies. Therefore, site-specific delivery approaches (both chemical, using small molecules or synthetically designed compounds, or biological, using antibodies) to inhibit succinate might be a good alternative in order to improve current cancer treatment. In any case, exploiting new and already known mechanisms by which succinate or other oncometabolites promote cancer that are specific in tumors and so not common with normal cells would be key to not only improve cancer patients survival, but also reduce side effects produced with these treatments. Following this concept, novel therapeutic approaches in which succinyl-CoA synthetase is inhibited might also be a good idea. In order to avoid side effects far from the tumor, a prodrug synthesis that targets specific cancer proteins or features could be an interesting option to make the drug action restricted to the tumor vicinity [213,214]. With this approach, it may be possible to broaden the clinical benefits for the Warburg-based treatments that might target SDH overexpressing tumors or any other tumors whose succinate expression is clearly upregulated. Moreover, it may also be interesting to study the synergetic effect of these inhibitors in combination with other current treatments such as antiangiogenic drugs or specific pathway inhibitors (i.e., JAK-STAT, PI3K-Akt). Considering all the presented data so far, we believe that targeting succinate production in cancer cells with Warburg metabolism may be beneficial to improve cancer treatment.

In this review, the Warburg effect and the main approaches to target it have been briefly summarized, and the role of succinate on cancer metabolism explained. In addition, the identification of succinate as a therapeutic target is suggested for consideration in future studies, and different approaches to do so have been presented.

## Data Availability

Not applicable.
